# Methacholine-Induced Variations in Airway Volume and the Slope of the Alveolar Capnogram Are Distinctly Associated with Airflow Limitation and Airway Closure

**DOI:** 10.1371/journal.pone.0143550

**Published:** 2015-11-23

**Authors:** Laurent Plantier, Sylvain Marchand-Adam, Laurent Boyer, Camille Taillé, Christophe Delclaux

**Affiliations:** 1 INSERM UMR 1152, Labex Inflamex, Paris, France; 2 Université Paris Diderot, PRES Sorbonne Paris Cité, Paris, France; 3 Assistance-Publique-Hôpitaux de Paris, Hôpital Bichat-Claude Bernard, Service de Physiologie-Explorations Fonctionnelles, DHU FIRE, Paris, France; 4 Hôpital Bretonneau, Service de Pneumologie, Tours, France; 5 Université François Rabelais, Tours, France; 6 INSERM U955, Créteil, France; 7 Université Paris-Est, Créteil, France; 8 Assistance-Publique-Hôpitaux de Paris, Hôpital Henri Mondor, Service de Physiologie-Explorations Fonctionnelles, Créteil, France; 9 Assistance-Publique-Hôpitaux de Paris, Hôpital Bichat-Claude Bernard, Service de Pneumologie A, Paris, France; 10 Assistance-Publique-Hôpitaux de Paris, Hôpital Européen Georges Pompidou, Service de Physiologie-Explorations Fonctionnelles, Paris, France; 11 Université Paris Descartes, Paris, France; 12 INSERM CIC Plurithématique 9201, Paris, France; University of Dundee, UNITED KINGDOM

## Abstract

Mechanisms driving alteration of lung function in response to inhalation of a methacholine aerosol are incompletely understood. To explore to what extent large and small airways contribute to airflow limitation and airway closure in this context, volumetric capnography was performed before (n = 93) and after (n = 78) methacholine provocation in subjects with an intermediate clinical probability of asthma. Anatomical dead space (VDaw), reflecting large airway volume, and the slope of the alveolar capnogram (slope3), an index of ventilation heterogeneity linked to small airway dysfunction, were determined. At baseline, VDaw was positively correlated with lung volumes, FEV_1_ and peak expiratory flow, while slope3 was not correlated with any lung function index. Variations in VDaw and slope3 following methacholine stimulation were correlated to a small degree (R^2^ = -0.20). Multivariate regression analysis identified independent associations between variation in FEV_1_ and variations in both VDaw (Standardized Coefficient-SC = 0.66) and Slope3 (SC = 0.35). By contrast, variation in FVC was strongly associated with variations in VDaw (SC = 0.8) but not Slope3. Thus, alterations in the geometry and/or function of large and small airways were weakly correlated and contributed distinctly to airflow limitation. While both large and small airways contributed to airflow limitation as assessed by FEV1, airway closure as assessed by FVC reduction mostly involved the large airways.

## Introduction

Non-specific bronchial hyperresponsiveness (BHR) is defined by an exaggerated decrease in forced expiratory flows in response to inhalation of agents inducing contraction of airway smooth muscle. Airway smooth muscle is present along the whole length of the bronchial tree [1]. The muscarinic agonist methacholine is the most commonly used such agent. BHR is considered a feature of bronchial asthma, although it is not specific to asthma since the prevalence of BHR is approximately twice that of asthma in the general population [2][3]. Better understanding of the factors underlying methacholine-induced airflow reductions may lead to refinements in bronchial challenge testing.

Because methacholine drives smooth muscle contraction in vitro and induces reductions in airway caliber that are detectable in vivo [4], and because subjects with BHR have an increased variability of peak expiratory flow rate as compared to subjects without BHR suggesting increased ease of airways to constrict [5], it is recognized that excessive methacholine-induced airway smooth muscle constriction and subsequent changes in airway geometry play key roles. Several lines of evidence, however, suggest that airway responses to methacholine are complex.

Earlier studies consistently show poor correlations between reductions in forced expiratory flows induced by methacholine inhalation and changes in airway resistance as measured during tidal ventilation, regardless of whether resistance was measured by plethysmography [6], forced oscillations [7][8], or the interrupter technique [9]. This discrepancy stresses that decreases in the forced expiratory volume in 1 second (FEV_1_) can be related to either reduction in the calibre of large and small airways, thus increasing airway resistance, or to focal airway closure which may decrease mobilizable lung volumes without inducing detectable changes in airway resistance, or to changes in elastic lung recoil. Airway imaging studies show either absent or poor correlations between changes in FEV_1_ and changes in airway calibre assessed by computed tomography (CT) scanning [10], while some studies demonstrate focally distributed paradoxical methacholine-induced bronchodilation [11], the latter finding supported by increases in Bohr’s anatomical dead space in asthmatics following methacholine provocation [12]. Measurements of ventilation distribution using ventilation scintigraphy and SPECT imaging [13], positron emission tomography (PET) imaging [14], Xe-enhanced CT scanning [15] and ^3^He-enhanced MRI [16] suggest that focal airway closure is a key feature of methacholine-evoked responses. Finally, computed tomography measurements of air trapping [17] or oscillometry-derived indices of small airway function [18][19][20] show that dysfunction of small airways is a feature of methacholine-induced responses in asthmatics.

Overall, available evidence suggests that methacholine-induced airflow reduction is associated with alterations in the geometry and function of both large and small airways. It remains to define, however, to what extent constriction of large and small airways contribute to airflow limitation and airway closure. To address this question, we used volumetric capnography to assess the strength of relationships between, on the one hand, variations in Fletcher’s dead space, an indicator of large airway volume [21][22], and variations in the slope of the alveolar capnogram, an index associated with small airway dysfunction [23][24], and on the other hand, with variations in FEV_1_ assessing airflow reduction and variations in forced vital capacity (FVC) assessing airway closure.

The slope of the alveolar capnogram reflects ventilation/perfusion mismatching in the distal lung due to regional variations in ventilatory time constants or perfusion. Because of the lack of effects of methacholine on the vasculature of the respiratory system [25][26], we assumed that the probability that alterations in lung perfusion determine variations in the slope of the alveolar capnogram in subjects with mild symptoms and without previous respiratory disease would be very low. Because slope3 is associated with the extent of small airway disease in resected lungs [23][24], as well as with disease severity in conditions where alterations of small airways play key roles, such as COPD [27] or cystic fibrosis [28], and because Slope3 is associated with airway resistance in humans [29], this index has been proposed as an indicator of small airway dysfunction.

## Materials and Methods

### Study design

Subjects eligible for enrollment into the study were patients over 18 years of age referred for bronchial provocation testing to the lung function department of a university hospital in Paris, France, because of suspected asthma as appreciated by the referring physician. Patients were included if they had no history of previous chronic respiratory disease, had not taken inhaled or oral steroids for three weeks prior to the visit, and had normal lung function at rest. Internal review board (IRB) approval was obtained (CEPRO, ethics committee of the French Language Pulmonology Society, #2013–011). In accordance with French law with regard to research into common clinical practice (Law #2004–806), subjects gave oral informed consent and were handed an explanatory leaflet, and their participation to the study was recorded in their clinical file. Participant consent was also recorded by the investigator in the main study folder. IRB approval was obtained for the consent procedure. After baseline forced spirometry, patients with normal lung volumes and FEV_1_/FVC ratio were invited to participate in the study. Plethysmography was performed before capnography and methacholine provocation testing. Volumetric capnography was performed before provocation testing and immediately after the last spirometry measures, less than 2 minutes after the last dose of methacholine, before albuterol was given. The presence of chronic cough (cough present over 3 or more consecutive months), exertional dyspnea (mMRC scale 1 or more), paroxysmal dyspnea or wheezing was recorded.

### Lung function testing at baseline

Forced spirometry and plethysmographic measurement of lung volumes and airway resistances were performed on the Jaeger Masterscreen system (Carefusion, Rolle, Switzerland), according to European Respiratory Society guidelines [30]. Lung function was considered normal if FEV_1_ and FEV_1_/VC were greater than the lower limit of normal as described by the mean predicted value– 1.64 standard deviation according to the ECCS1993 reference equations for European populations [31].

### Methacholine provocation testing

Provocation testing was performed using the automated APS tidal ventilation aerosolization system (Jaeger) and methacholine chloride in phosphate buffered saline (Assistance Publique-Hôpitaux de Paris, France). The initial dose was 100 μg (0.625 μmol), followed by doses of 0.1mg, 0.3mg (1.250020μmol), 0.4mg (2.5 μmol) and 0.8mg (5μmol). Forced spirometry was performed 90 sec after each dose. Increasing doses of methacholine were administered until FEV_1_ fell by 20% in comparison to the baseline value, or until a total dose of 1.6 mg (10 μmol) was delivered. BHR was defined as a FEV_1_ decrease ≥20% in response to a maximal methacholine dose of 1.6 mg [32]. Airway reactivity was quantitated in all subjects by the FEV_1_-dose-response slope (DRS-FEV_1_) and the FVC-dose-response slope (DRS-FVC), defined by the respective reduction in FEV_1_ and FVC expressed as a percentage divided by the methacholine dose. The dose inducing a 20% fall in FEV1 (PD_20_FEV_1_) was calculated by linear interpolation in subjects with BHR.

### Volumetric capnography

Volumetric capnography was performed during exhalations from total lung capacity (TLC) using the FE141 differential pressure spirometer and the ML-206 gas analyser, both from AD Instruments (Oxford, UK). Signals were synchronized and digitized at a sampling frequency of 500 Hz using LabChart software v7.3.1 (AD Instruments). Patients were connected to the capnography apparatus through a non-resistive filter (PF30, Pall, Fribourg, Switzerland). Every five tidal breaths, patients were instructed to inhale to TLC, and to immediately exhale passively. Subjects were asked to maintain their expiratory flow at the level observed during tidal breathing by visual feedback of flow measurements. Expiratory volume and the expired fraction of CO_2_ (FeCO_2_) were exported to a spreadsheet and airway volume (VDaw) was calculated according to Fletcher’s method for determination of anatomical dead space using equal area determination [33]. To assess ventilation heterogeneity related to small airway dysfunction [23][24][27][28], the slope of the alveolar (third) phase of the volumetric capnogram (slope3) was determined [33]. Five consecutive exhalations from TLC were acquired. Because distribution of VDaw and slope3 values was not normal in all subjects, the median airway volume and phase 3 slope values were retained for analysis. Changes in airway volume and ventilation heterogeneity were expressed relative to the total methacholine dose in mg (ΔVDaw/Mch and ΔSlope3/Mch). In addition, airway volume and slope 3 were measured during the first five tidal breaths taken before the first inspiration to TLC, and their changes relative to the methacholine dose were calculated as described above (ΔVDaw_tidal_/Mch and ΔSlope3_tidal_/Mch).

### Statistical analysis

Data were expressed as mean +/- standard deviation, with the exception of PD_20_ which was expressed as mean and range because it is log-distributed. Relationship between baseline capnographic indices and baseline lung function parameters were determined by linear regression. To assess the association between capnographic indices with airflow reduction, we explored relationships between the FEV_1_ and FVC dose-response slopes (dependent variables) and both the baseline values in VDaw and slope3 and the fractional changes induced by methacholine in these indices (ΔVDaw/Mch and ΔSlope3/Mch) expressed as %/mg methacholine (independent variables). Univariate linear regression was performed, followed by multiple regression testing variables showing a significant association by univariate analysis. Differences between subjects without and with BHR were determined by student’s t-test for continuous variables and the Chi-square test for categorical variables. All statistical analyses were performed with Statview 5.0 software (SAS institute, Cary, North Carolina, USA). A p-value <0.05 was considered significant. Because multivariate analysis with 2 variables was scheduled, we aimed to recruit at least 20 patients with BHR. Because the expected prevalence of BHR is close to 25% among patients referred for provocation testing at our center, we thus planned to recruit at least 80 subjects. Original data are available as an Excel file in the online supplement (S1 Original Data).

## Results

### Baseline characteristics of subjects

A total of 93 subjects were included into the study between June 3^rd^, 2013 and October 8^th^, 2014 (Table 1). Of these, 25 (27%) had BHR defined by PD_20_FEV_1_ ≤ 1.6mg, with a mean PD_20_ of 0.69 mg (range: 0.09–1.52). Patients with BHR showed signs of airflow limitation at baseline in comparison with those without BHR, as indicated by lower FEV_1_, FEF_25-75_, FEF_25-75_/FVC, FEV_1_/FVC, and higher airway resistance. By contrast, neither airway volume nor slope3 were different at baseline. Symptoms were not different in subjects with BHR in comparison with subjects without BHR, consistent with an intermediate pre-test probability of asthma for all patients.

**Table 1:** Characteristics of subjects before methacholine testing. FEV1: Forced expiratory volume in 1 second. FVC: Forced vital capacity. FEF25-75: Mean forced expiratory flow between 25% and 75% of FVC. PEF: Peak expiratory flow. TLC: Total lung capacity. RV: Residual volume. Raw: Airway resistance. sRaw: Specific airway resistance. VDaw: Airway volume. Slope3: Slope of the alveolar capnogram.

	No BHR (n = 68)	BHR (n = 25)	All (n = 93)
Gender: male	42%	32%	40%
Age (years)	41 +/- 14	45 +/- 15	42 +/- 14
Height (cm)	169 +/- 8	166 +/- 9	168 +/- 9
Body Mass Index	24 +/- 4	27 +/- 6*	25 +/- 6
Tobacco (Pack-years)	7 +/- 14	4 +/- 10	6 +/- 13
FEV1 (% pred)	100 +/- 14	92 +/- 16*	97 +/- 15
FVC (% pred)	100 +/- 18	103 +/- 19	101 +/- 18
FEV1/FVC	0.82 +/- 0.08	0.78 +/- 0.13	0.81 +/- 0.1
FEF25-75 (% pred)	85 +/- 24	61 +/- 18 ***	79 +/- 25
FEF25-75/FVC	0.86 +/-0.27	0.63 +/-0.24 **	0.80 +/- 0.28
PEF (% pred)	104 +/- 17	91 +/- 24	101 +/- 18
TLC (% pred)	102 +/- 14	93 +/- 31	108 +/- 14
RV (% pred)	100 +/- 26	102 +/- 23	101 +/- 25
RV/TLC (%)	31 +/- 8	37 +/- 17	32 +/- 12
Raw (kPa.L^-1^.s^-1^)	0.24 +/- 0.1	0.36 +/- 0.27*	0.27 +/- 0.15
VDaw (mL)	209 +/- 69	185 +/- 69	202 +/- 69
VDaw/TLC (mL/L)	35 +/- 11	33 +/- 8	34 +/- 10
Slope3 (%CO_2_/L)	0.63 +/- 0.27	0.70 +/- 0.29	0.65 +/- 0.28
Cough	65%	60%	63%
Wheezing	28%	40%	32%
Paroxysmal dyspnea	43%	60%	48%
Exertional dyspnea	37%	40%	38%

*: p < 0.05,

**: p<0.001,

***: p<0.0001 between subjects with and without BHR.

### VDaw, but not Slope3, was associated with lung volumes

Associations between baseline capnographic indices and baseline lung function were explored by linear regression. As shown in Table 2, VDaw was positively associated with TLC, FVC, FEV_1_, the peak expiratory flow rate (PEF) and FEF25-75, consistent with the expected correlation between VDaw and lung size, but neither with residual volume (RV) nor the maximum midexpiratory flow (FEF_25-75_), nor FEV1/FVC. By contrast, no significant association was observed between slope3 and any lung function index.

**Table 2:** Correlation between baseline lung function parameters and baseline airway volume (VDaw) and the slope of the alveolar capnogram (slope3) in 93 subjects. Linear regression was performed with VDaw and slope3 as dependent variables.

	VDaw		slope3	
	R^2^	p	R^2^	p
FEV1 (L)	0.16	<0.001	0.01	0.8
FVC(L)	0.26	<0.0001	0.07	0.52
PEF (L.s^-1^)	0.3	<0.0001	4E-04	0.8
FEF25-75 (L.s^-1^)	0.06	0.02	0.07	0.24
RV (L)	2E-04	0.91	0.05	0.17
TLC (L)	0.21	<0.0001	0.02	0.27
Raw (kPa.L^-1^.s^-1^)	0.1	0.12	0.03	0.26
FEV1/FVC	0.003	0.65	0.012	0.39

### Decreased airway volume and increased ventilation heterogeneity were independently associated with methacholine-induced changes in FEV_1_

**Fig 1:**
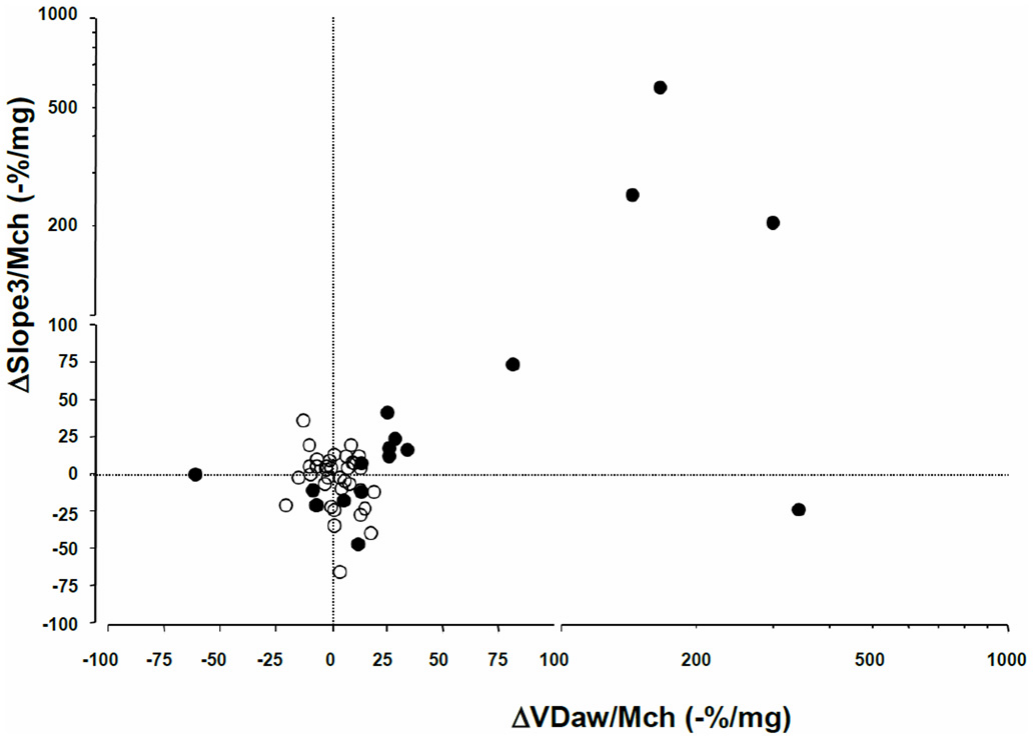
Relationships between methacholine-induced variations in airway volume and the slope of the alveolar capnogram, in patients without BHR (empty circles) and in patients with BHR (filled grey circles). Scales are linear to a value of 100 and exponential thereafter. ΔVDaw / Mch: Fractional change in aiway volume reported to the methacholine dose, expressed as -% / mg. Δslope3 / Mch: Fractional change in the slope of the alveolar capnogram reported to the methacholine dose, expressed as % / mg.

Post-methacholine capnography data were available for 78 subjects (n = 55 without BHR and n = 23 with BHR). To characterize relationships between variations in capnographic variables and airflow limitation, univariate and multivariate regression analyses were performed. FEV_1_ and FVC dose-responses were strongly correlated (R^2^ = 0.79, p<0.0001). Baseline VDaw and ΔVDaw/Mch were not correlated, nor were baseline Slope3 and ΔSlope3/Mch. ΔVDaw/Mch and ΔSlope3/Mch were correlated, although the strength of the correlation was weak (R^2^ = -0.20, p<0.001, Fig 1). There was no correlation between DRS-FEV1 and either baseline VDaw/TLC (R^2^ = 0.002 p = 0.70) or baseline Slope3 (R^2^<0.0001, p = 0.54), nor between DRS-FVC and either baseline VDaw/TLC (R^2^<0.0001 p = 0.97) or baseline Slope3 (R^2^<0.0001, p = 0.47).

**Fig 2:**
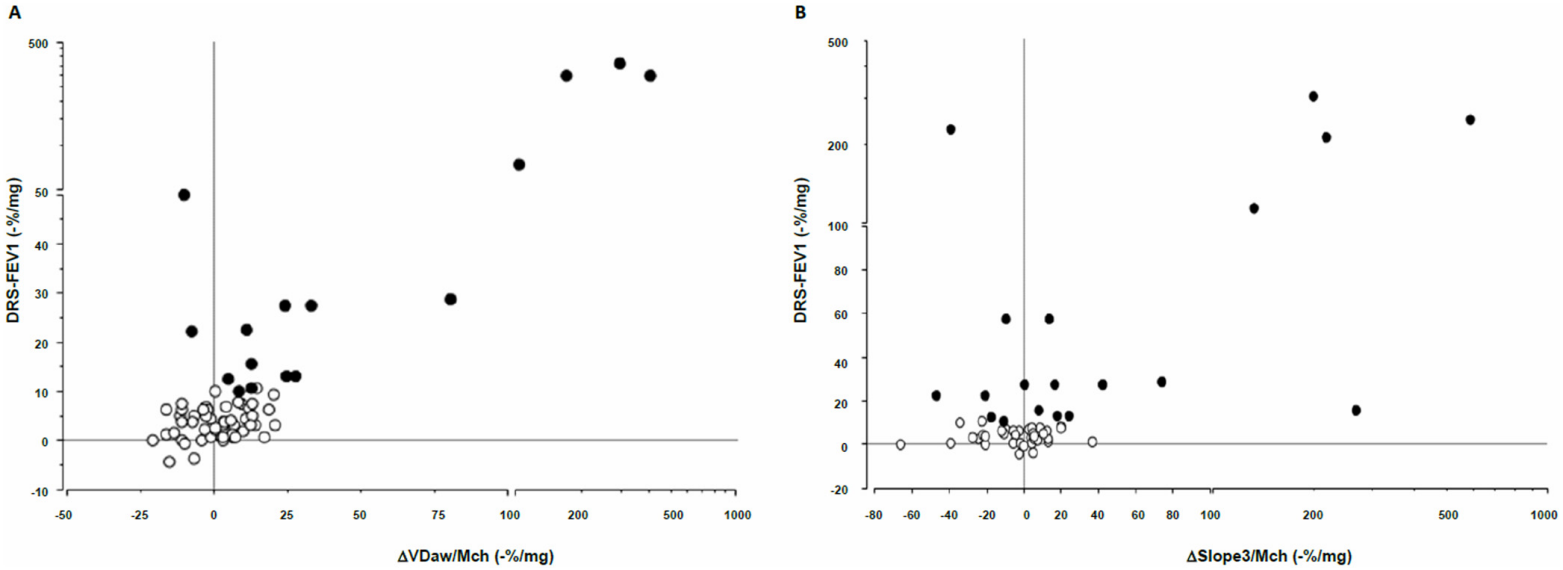
Relationships between methacholine-induced variations in volumetric capnography variables and the FEV1 dose-response curve in patients without BHR (empty circles) and in patients with BHR (filled grey circles). A: Variations in airway volume (VDaw). B: Variation in the slope of the alveolar capnogram (slope3). Scales are linear to a value of 100 and exponential thereafter. ΔVDaw / Mch: Fractional change in aiway volume reported to the methacholine dose, expressed as -% / mg. Δslope3 / Mch: Fractional change in the slope of the alveolar capnogram reported to the methacholine dose, expressed as % / mg.

**Fig 3:**
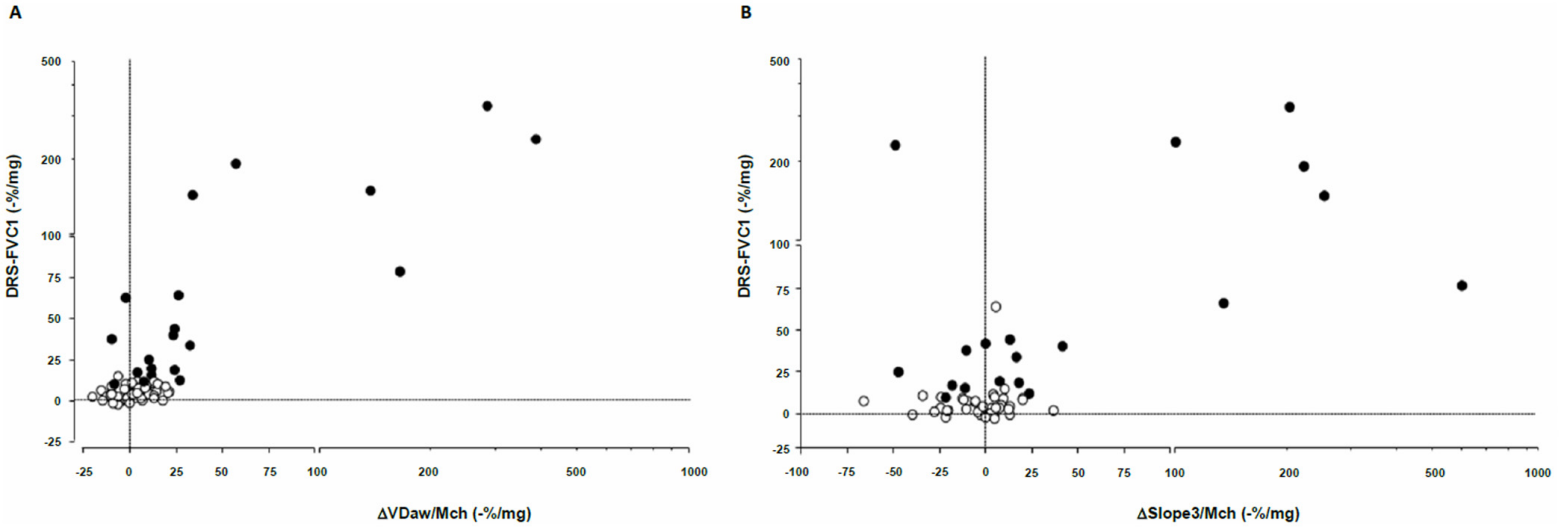
Relationships between methacholine-induced variations in volumetric capnography variables and the FVC dose-response curve in patients without BHR (empty circles) and in patients with BHR (filled grey circles). A: Variations in airway volume (VDaw). B: Variation in the slope of the alveolar capnogram (slope3). Scales are linear to a value of 100 and exponential thereafter. ΔVDaw / Mch: Fractional change in aiway volume reported to the methacholine dose, expressed as -% / mg. Δslope3 / Mch: Fractional change in the slope of the alveolar capnogram reported to the methacholine dose, expressed as % / mg.

Table 3 shows univariate relationships between the methacholine dose-response of FEV_1_ and FVC, and the methacholine dose-response of capnographic variables. The DRS-FEV_1_ was strongly associated with reductions in airway volume in subjects both without and with BHR (Fig 2A). The relationship between variations in Slope3 adjusted for methacholine dose and DRS-FEV_1_, although significant, was weaker (Fig 2B). Likewise, univariate associations were observed between the DRS-FVC and both reduction in VDaw (Fig 3A) and increases in slope3 (Fig 3B). By contrast, neither baseline VDaw nor baseline Slope3 were associated with methacholine-induced reductions in FEV1 or FVC. When analysis was restricted to patients with BHR, significant association remained between ΔVDaw/Mch and both DRS-FEV_1_ (R^2^ = 0.56, p<0.0001) and DRS-FVC (R^2^ = 0.66, p<0.0001), and between ΔSlope3/Mch and DRS-FEV1 (R^2^ = 0.33, p = 0.01).

**Table 3:** Univariate correlation between the FEV1 and FVC dose-response, baseline airway volume and Slope3, and Mch-induced variations in Vdaw and Slope3. Linear regression was performed with FEV1 and FVC DRS as dependent variables.

	All (n = 78)	
	R^2^	p
DRS-FEV1		
Baseline VDaw	0.02	0.7
Baseline Slope3	0.06	0.54
ΔVDaw/Mch	0.62	<0.0001
ΔSlope3/Mch	0.42	<0.0001
DRS-FVC		
Baseline VDaw	<0.01	0.97
Baseline Slope3	0.09	0.48
ΔVDaw/Mch	0.69	<0.0001
ΔSlope3/Mch	0.23	<0.0001

Additional analyses tested whether capnographic parameters measured during tidal breathing correlated with DRS-FEV1 and DRS-FVC. Analysable three-phase capnograms were obtained in 49 patients. In contrast with measurements made from TLC, we did not observe any significant association between ΔVDaw_tidal_/Mch and either DRS-FEV1 (p = 0.96, R^2^<0.0001) or DRS-FVC (p = 0.87, R^2^ = 0.001), nor between ΔSlope3_tidal_/Mch and either DRS-FEV1 (p = 0.53, R^2^ = 0.009) nor DRS-FVC (p = 0.51, R^2^ = 0.009).

Multivariate regression (Table 4) demonstrated the independent association of variations in both large airway volume and slope3 with FEV_1_ reduction; the regression coefficient for the model was 0.77. By contrast, while reductions in FVC were quite strongly associated with variation in airway volume, no independent association was observed between FVC and variations in slope3. When analysis was restricted to patients with BHR, significant association remained between ΔVDaw/Mch and both DRS-FEV_1_ (R^2^ = 0.65, p = 0.0003) and DRS-FVC (R^2^ = 0.81, p<0.0001), and between ΔSlope3/Mch and DRS-FEV1 (R^2^ = 0.33, p = 0.03).

**Table 4:** Multivariate analysis of relationships between the FEV1 and FVC dose-response and Mch-induced variations in capnographic variables. SC: Standardized coefficient.

	All (n = 78)	
	SC	p
DRS-FEV1		
ΔVDaw/Mch	0.66	<0.0001
ΔSlope3/Mch	0.35	<0.0001
DRS-FVC		
ΔVDaw/Mch	0.80	<0.0001
ΔSlope3/Mch	0.13	0.11

## Discussion

The main results of this study are 1) that methacholine-induced variations in airway volume and the slope of the alveolar capnogram were weakly associated and 2) that variations in airway volume were associated with reduction in both FEV1 and FVC, while 3) variations in the slope of the alveolar capnogram were associated with reduction in FEV1 but not FVC. These data suggest that, in the context of methacholine provocation, changes in airflow (FEV_1_) occur across proximal and distal airways, whereas changes in lung volume (FVC) are associated with changes in large airways only, suggesting closure of large airways.

As expected, baseline VDaw was highly correlated with indices of lung and airway size. Of note, the strength of the relationship between VDaw and the peak expiratory flow rate in our study was strikingly similar with that reported for radiographic measurements of tracheal size [34], consistent with the association between VDaw and the cross-sectional area of proximal airways measured by acoustic reflexion [21]. Conversely, VDaw was not associated with indices of small airway dysfunction such as decreased FEF_25-75_ and increased residual volume. Altogether, these data support the notion that VDaw mainly explores proximal large airways but not distal small airways. Although the delineation of proximal and distal airways in this context is imprecise, theoretical arguments suggest that airways with an internal diameter over 2 mm may be explored by VDaw [22].

The absence of a significant association between baseline proximal airway volume and airway responsiveness in the present study is consistent with our previous observation that tracheal volume is not associated with airway responsiveness in patients with nasal polyposis [35], and suggests that interindividual variation in the baseline volume of large airways is not an important determinant of BHR. This result contrasts with the known association between BHR and low forced expiratory flows at low lung volume, and in particular the FEF_25-75_/FVC ratio, which was interpreted as an indication for the involvement of anatomical variation of small airways in BHR [36]. This discrepancy suggests that proximal and distal airway sizes may not be strongly correlated in patients with respiratory symptoms. Based on theoretical grounds, the progressive reduction of airway calibre at each generation can be described by a single constant factor, the homothety ratio, due to the fractal nature of the bronchial tree [35]. Thus, one may hypothesize that patients with BHR could have a reduced homothety ratio, which would not impact the volume of proximal airways but would increase the propensity for distal airflow limitation to occur. Whether the hypothesized decrease in the homothety ratio could be related to underlying anatomy or related to a remodelling process, as suggested for COPD [37], remains to be studied.

The absence of a relationship between baseline Slope3 and airway responsiveness contrasts with findings where ventilation heterogeneity assessed by the multiple breath nitrogen washout technique at baseline was associated with the dose response ratio to methacholine, which is analogous to DRS-FEV_1_. In a group of 40 asthmatics, airway responsiveness was significantly associated with Scond, an index of ventilation heterogeneity at the level of conducting airways [38]. In another series of 19 asthmatics, airway responsiveness was correlated with Sacin, an index of ventilation heterogeneity in the diffusing regions of the lung [39]. A possible explanation for the lack of an association between baseline capnographic indices and airway responsiveness may be the high interindividual variability in the former [29].

Previous studies showed correlation between decrements of FEV1 and FVC under methacholine stimulation, suggesting that airway closure is a key factor in airflow reduction in this context, as well as an independent but likely weaker association of FEV1 decrements with reduction in the FEV1/FVC ratio consistent with a role for airway narrowing in non-occluded lung regions [40]. Furthering these studies, we observed that decreases in FVC were strongly associated with decreases in both FEV_1_ and VDaw, but not with the slope of the alveolar capnogram which reflects ventilation/perfusion mismatching in lung regions participating to ventilation but does not provide information about lung regions excluded from ventilation. These data support a model where complete closure of large airways reduces FVC and FEV_1_ almost proportionally, as suggested by the formation of large and complete ventilation defects in the lungs of asthmatics, the topography of which is consistent with segmental or subsegmental localization [15][16][41]. Whether closure of large airways occurs focally, independent of small airway narrowing, or in association with distal airway closure propagating up the airway tree, remains to clarify. The hypothesis that large airway closure occurs focally is supported by imaging studies directly showing focal stenosis of large airways following methacholine provocation [42]. Conversely, studies relying on the combination of computational modelling and PET imaging of ventilation defects suggest that narrowing of both large and small airway drives the constitution of ventilation defects. Particularly, Tgavalekos and colleagues used a model combining PET and oscillatory mechanics to analyse data from 6 asthmatics in the aim of inferring the site of airway responses, and showed that either constriction of small (diameter<2.4mm) airways alone, or simultaneous constriction of large and small airways could explain both ventilation defects and mechanical alterations induced by inhaled methacholine [43]. Multiple breath nitrogen washout studies in normal subjects and asthmatics showed association between airway closure and Scond [44][45]. Overall, available data suggest that a combination of large and small airway responses drives alterations in lung physiology in the setting of methacholine provocation.

Methacholine-induced variations in VDaw and in the slope of the alveolar capnogram were weakly associated in our study. In addition, the association of increases in slope3 with decreases in FEV1, consistent with participation of small airway contraction to methacholine-induced airflow limitation, was independent of variations in VDaw which reflects large airway geometry. This result suggests that variation in these two capnographic indices, and thus presumably involvement of large and small airways in the response to methacholine, may be driven in part by distinct mechanisms. This hypothesis is supported by a similar observation in the context of exacerbated asthma, where acinar ventilation heterogeneity and conducting airway heterogeneity measured by the multiple breath nitrogen washout technique were independent from each other [46]. Interestingly, in line with the differential expression of cholinergic receptors in large and small airways [47], experimental studies suggest that the responses of proximal and distal airways to a cholinergic stimulus may be different in dogs [48], rats [49] and mice [50].

Our results are consistent with previous work showing increases in KPIv, an index integrating changes in both the second and third phases of the volumetric capnogram, in asthmatic children following induced bronchoconstriction [51]. Importantly, capnography during exhalation from TLC in our work provided information complementary to previous studies where the impact of methacholine on capnography variables was determined during tidal breathing [52][22]. In particular, although we showed a tight relationship between changes in airway volume measured during tidal volume and FEV1 variation, such an association was not evidenced during tidal breathing in normal subjects [52], a finding replicated in our study. Measurements from TLC allow for the quantitation of the volume of the whole bronchial tree, and can thus detect changes related to localized airway closure, as well as to generalized bronchoconstriction. By contrast, measurements made during tidal breathing only explore airway segments participating in tidal ventilation, and may thus be biased by the preferential exploration of airway segments that are either unaffected by methacholine exposure, or subjected to methacholine-induced bronchodilation [11]. Such sampling bias may explain why variations in VDaw were not associated with variations in the resistance of the respiratory system as measured by the force oscillation technique in asthmatics, thus raising the possibly misled hypothesis that alterations in the function of small airways played key roles in methacholine-evoked responses in these subjects [22].

Methodological limitations of our study must be kept in mind. All patients had respiratory symptoms cautioning against translation of results to asymptomatic subjects. Only subjects not receiving asthma medication and without signs of airflow obstruction at baseline were studied, thus precluding any conclusion regarding mechanisms of methacholine-induced airflow limitation in patients with moderate or severe asthma, or in treated patients. In addition, we ackowledge that, in the absence of a direct comparison between asthmatics and normal controls, our study could not contribute to the identification of capnographic patterns that may be of use for the diagnosis of asthma. The question whether bronchial inflammation was associated with capnographic indices and their variations under provocation was not addressed. Also, it must be acknowledged that determination of Fletcher’s dead space by volumetric capnography relies on modelization of the interface of atmospheric and alveolar gas at the start of expiration, and is an estimation of airway volume rather than a direct measurement [53]. In addition, inspiration to TLC during capnography integrates the bronchoprotective and bronchodilatory effect of deep inspiration. It is possible that the well-demonstrated lack of such effects of deep inspirations in most asthmatics [54] may explain part of our results. In particular, one may ask whether airway volume and slope3 measured after methacholine inhalation, but before the deep inspirations associated with spirometry, would have been associated with airflow limitation and airway closure. In any case, we believe that performing capnography after spirometry controlled for the effect of deep inspirations and allowed to study relationships between spirometric and capnographic parameters in a majority of subjects.

In conclusion, our results show that variations in capnographic indices reflecting large airway volume and small airway dysfunction are independently associated with methacholine–induced reductions in FEV_1_, indicating that both large and small airways contribute to airflow limitation in the context of methacholine provocation testing. This result supports the use of FEV1 as the outcome variable during methacholine provocation, as this parameters integrates alterations in the geometry and function of both proximal and distal airways. In addition, the observation that methacholine-induced reduction in FVC, which relates to airway closure, was associated with variation in VDaw, which reflects the volume of large airways, but not with variations in Slope3, which reflects small airway dysfunction, indicates that changes in FVC relate for a major part on the closure of large airways following methacholine stimulation.

## Supporting Information

S1 Original Data Data are supplied as a Microsoft Excel file. (XLS)
